# Differential physiological and biochemical modulation of two grapevine varieties by foliar zeolite application under Mediterranean summer stress conditions

**DOI:** 10.3389/fpls.2026.1833884

**Published:** 2026-05-20

**Authors:** Sandra Martins, Miguel Baltazar, Cátia Brito, Ana Monteiro, Zélia Branco, Ricardo Santos, Renato Dinis, Fábio Tavares, Sandra Pereira, Lia-Tânia Dinis

**Affiliations:** 1 CITAB - Centre for the Research and Technology of Agro-Environmental and Biological Sciences, University of Trás-os-Montes and Alto Douro (UTAD), Vila Real, Portugal; 2 Inov4Agro - Institute for Innovation, Capacity Building and Sustainability of Agri-Food Production, University of Trás-os-Montes and Alto Douro (UTAD), Vila Real, Portugal

**Keywords:** climate change, long-term resilience, particle film, sustainable strategies, Tinto Cão, Touriga Franca

## Abstract

**Introduction:**

The intensification of climate change poses significant challenges to viticulture, particularly in Mediterranean region. In this context, the development and implementation of sustainable practices that simultaneously address mitigation and adaptation are essential to ensure vineyard resilience. This study evaluated the combination of a short-term mitigation strategy, the foliar application of clinoptilolite zeolite, with a long-term adaptation approach based on varietal selection.

**Methods:**

The impacts of foliar zeolite applications on two-year-old grapevines (*Vitis vinifera L.*) of Touriga Franca (TF) and Tinto Cão (TC) varieties were evaluated through the assessment of leaf gas exchange, chlorophyll a fluorescence, primary and secondary metabolites, antioxidant capacity, and leaf histological parameters.

**Results:**

Zeolite application mitigated heat stress in both varieties by decreasing leaf temperature, enhancing photochemical efficiency, and supporting photoprotection. Variety-specific responses were also notable. TF exhibited improved electron transport, gas exchange, and higher accumulation of soluble sugars, proline, and proteins, reflecting enhanced physiological plasticity. In turn, TC displayed higher intrinsic phenolic content and antioxidant capacity, adopting a more conservative water-use strategy.

**Discussion:**

These findings highlight the combined role of short- and long-term agronomic practices to strengthen vineyard resilience under changing climate conditions.

## 1 Introduction

Viticulture faces increasing challenges due to the intensification of climate change, particularly in Mediterranean region (Lazoglou et al., 2024). Rising global average temperatures, together with more frequent and intense extreme climatic events and altered precipitation patterns, exert strong pressure on wine-producing areas such as the renowned Douro Demarcated Region (DDR) (Fonseca et al., 2023; Lazoglou et al., 2024; Yuan et al., 2024). Although *Vitis vinifera* L. exhibits considerable adaptive capacity to cope with combined abiotic stresses, the intensification of these climatic constraints can impair essential plant physiological and biochemical functions (Leeuwen and Destrac-Irvine, 2017; van Leeuwen et al., 2019; Brito et al., 2024).

Specifically, under these conditions, photosynthesis is limited by stomatal closure and damage to the photosynthetic machinery, thereby reducing carbon assimilation and disrupting the synthesis and translocation of photoassimilates (Bernardo et al., 2018; Gambetta et al., 2020). The overproduction of reactive oxygen species (ROS) triggers oxidative stress, which damages cellular function, leads to protein degradation, and impairs overall metabolic balance (Carvalho et al., 2024). In response, grapevines activate a range of protective mechanisms, including the biosynthesis and accumulation of phenolic compounds with antioxidant properties, as well as osmolytes such as proline, which support osmotic adjustment and cellular stabilization under adverse environmental conditions (Gambetta et al., 2020; Dinis et al., 2024). These physiological and metabolic adjustments have direct implications for grape yield and wine quality (Dinis et al., 2024).

It is therefore essential to study and implement sustainable practices that contribute to both the mitigation and adaptation of the negative effects of climate change in viticulture, ensuring the long-term resilience and sustainability of wine-producing regions (van Leeuwen et al., 2019). These strategies can be broadly classified into short- and long-term approaches (Baltazar et al., 2025a). Short-term measures include immediate adjustments in vineyard management practices aimed at alleviating stress under current climatic conditions, whereas long-term strategies involve structural changes designed to enhance the long-term resilience of viticultural systems (Naulleau et al., 2021; Santos et al., 2021; Baltazar et al., 2025a).

In this regard, the application of foliar particle films represents an effective short-term strategy. Clinoptilolite zeolites, naturally occurring crystalline aluminosilicates, exhibit a high cation exchange capacity (CEC), as well as strong adsorption and water retention properties (Cataldo et al., 2021; Grifasi et al., 2025). Consequently, their foliar application has the potential to reduce leaf temperature, improve plant water status, and enhance water use efficiency under abiotic stress conditions, leading to higher plant growth and yield (Cataldo et al., 2021; Conversa et al., 2024; Rotondi et al., 2025). In turn, varietal selection constitutes a long-term strategy aimed at ensuring the sustainability of vineyards through the use of more resilient grapevine varieties (van Leeuwen et al., 2019). Touriga Franca (TF) and Tinto Cão (TC) are two red grapevine varieties of great importance in the DDR, each exhibiting distinct agronomic and physiological traits (Brito et al., 2024). TF is distinguished by its regular productivity and high qualitative potential (Magalhães, 2015) whereas TC, despite its low productivity, shows excellent adaptation to thermal and water stress and has been identified as a promising variety under future warming scenarios (Moutinho-Pereira et al., 2007; Brito et al., 2024).This study aims to evaluate the effects of foliar zeolite application on TF and TC grapevine varieties, both grafted onto the 1103 Paulsen (1103 P) rootstock. Although the effects of foliar zeolite application on grapevines have been previously reported (Petoumenou, 2023; Allegro et al., 2024; Valentini et al., 2025), and varietal differences in stress response are well documented (Moutinho-Pereira et al., 2007; Brito et al., 2024; Baltazar et al., 2025a), to the best of our knowledge, no studies have integrated these two approaches within a single experimental framework. Therefore, this study offers an integrated evaluation of the synergistic effects of foliar zeolite application and varietal selection on grapevine physiological, biochemical, and anatomical traits. Considering these points, we hypothesized that: (i) foliar application of zeolite enhances grapevine physiological performance under drought; (ii) the effects of zeolite application are variety-dependent; and (iii) the combination of foliar zeolite application with varietal selection enhances vineyard resilience to climate change. This integrated approach provides a comprehensive framework and proposes an innovative and sustainable strategy to enhance vineyard resilience and competitiveness under changing climatic conditions.

## 2 Material and methods

### 2.1 Plant material and experimental design

**Figure 1:**
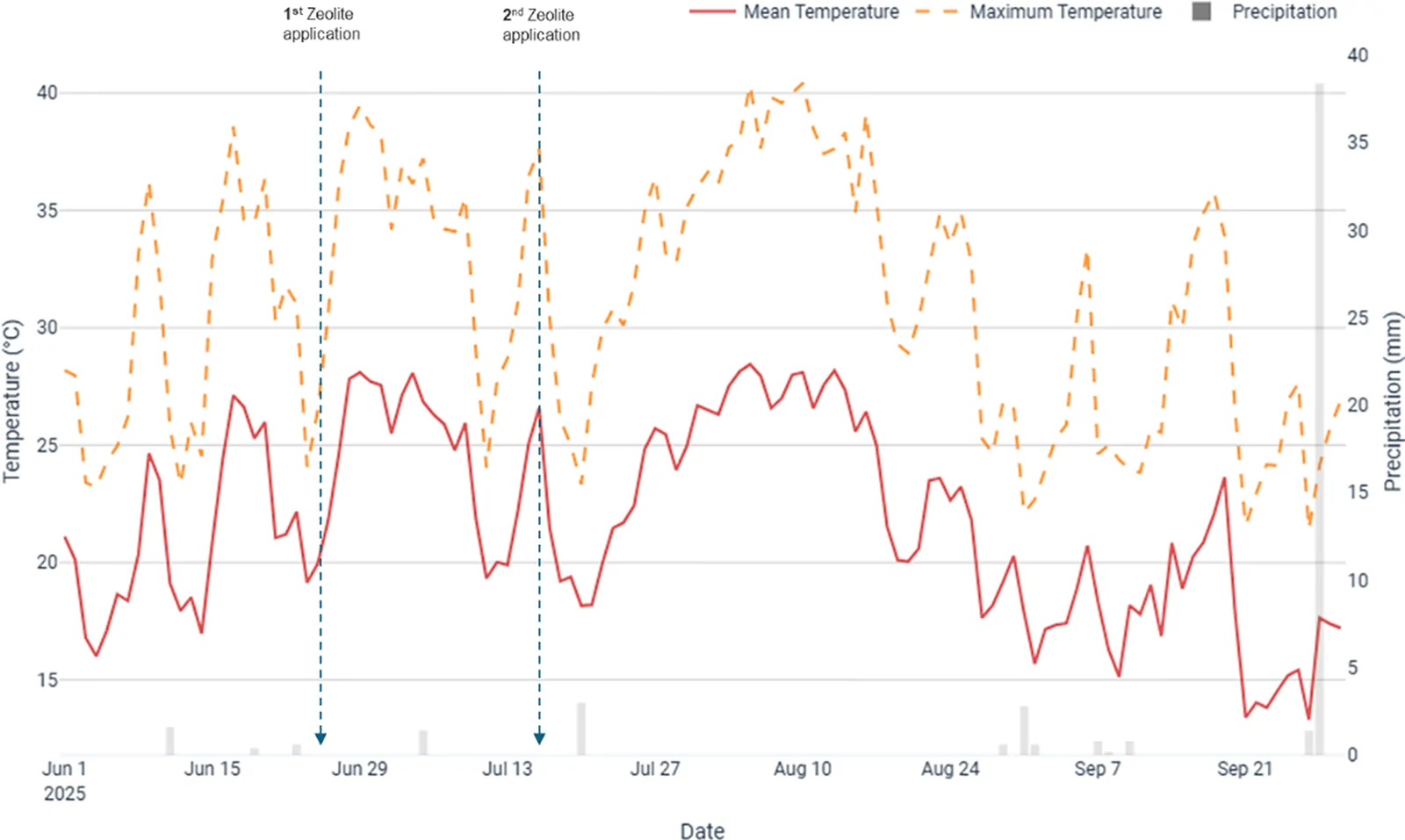
Daily average and maximum temperature patterns with precipitation values from June to September 2025. The dotted lines indicate the dates of zeolite application (26 June and 16 July 2025).

The experiment was conducted at the University of Trás-os-Montes e Alto Douro (41°17′14.8″ N 7°44′14.8″ W, 500 m above sea level), Baixo Corgo sub-region of the DDR, Vila Real, northern Portugal, from June to September 2025. This region exhibits typical Mediterranean summer conditions, with persistently high air temperatures, recurrent extreme maximum temperature events, and very low and irregular rainfall. **Figure 1** illustrates the monthly total precipitation and mean air temperature recorded during the experimental period at the meteorological station of the University of Trás-os-Montes e Alto Douro.

The experimental trial was carried out using two grapevine (*Vitis vinifera* L.) varieties, TF and TC, both two-year-old vines grafted onto 1103P rootstock. Plants were grown outdoors in 15 L pots containing a silty loam soil amended with peat and perlite. The soil was sampled from the surface horizon (0–20 cm), air-dried, and passed through a 5 mm sieve prior to use. The experimental design comprised four groups with five replicates per treatment. For each variety, two treatments were established, foliar application of zeolites and a non-treated control, resulting in the following experimental groups: TF Control, TF Zeolites, TC Control, and TC Zeolites. Clinoptilolite zeolites were applied at 3.5 kg ha^-^¹, following the manufacturer’s recommendation (DRYCEL, ZEOCEL Portugal Lda). An aqueous suspension containing zeolites and 0.1% (v/v) Tween 20 was prepared to enhance leaf adhesion and ensure uniform spraying. Applications were performed twice during the summer period, on June 26 and July 16, 2025. The physicochemical properties of zeolites, as provided by the manufacturer, are summarized in **Table 1**. Control plants were sprayed with water, following the same application method used for the zeolite treatment.

**Table 1:** Main physicochemical properties of the zeolites used in the experimental design, as provided by the manufacturer.

Clinoptilolite zeolites	
(Ca, K_2_, Na_2_, Mg)_4_Al_8_Si_40_O_96_ 24H_2_O	
Chemical composition	
SiO_2_	72.4%
CaO	4.44%
K_2_O	3.12%
Fe_2_O_3_	1.60%
Ion-exchange capacity	
Ca^2+^	0.64-0.98 mol kg^-1^
K^+^	0.2-0.45 mol kg^-1^
Mg^2+^	0.06-0.19 mol kg_-1_
Na^+^	0.01-1.50 mol kg^-1^
CEC	1.70-2.10 mol kg^-1^
Physical properties	
Porosity	24-32%
Pore diameter	0.4 nm
Density	300–600 kg m^-3^

**Figure 2:**
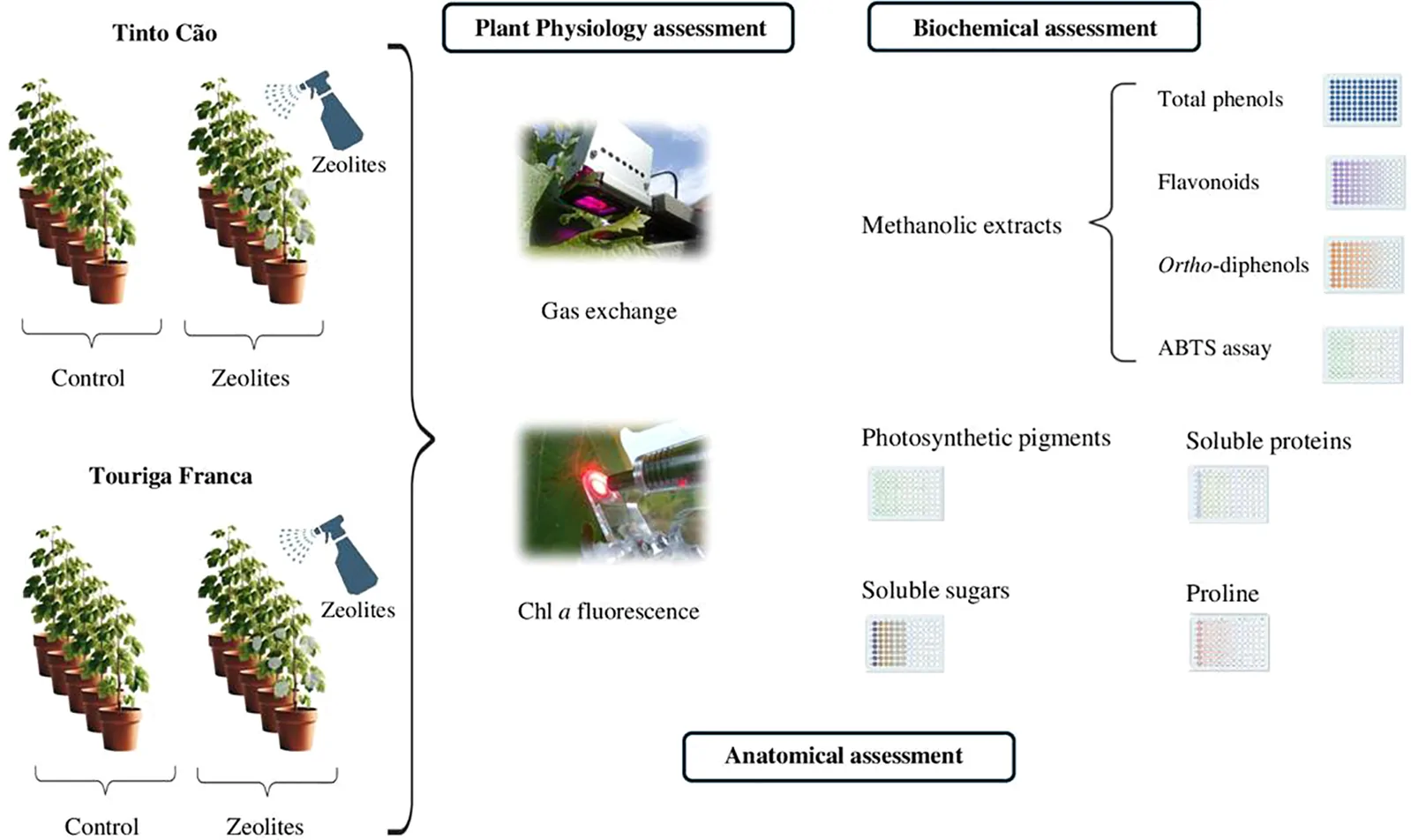
Schematic representation of the experimental design and applied methodologies.

Throughout the experiment, physiological and biochemical parameters were systematically monitored (**Figure 2**). Leaf gas exchange and chlorophyll *a* fluorescence were measured *in situ* at 20, 42, and 70 days after the first application (DAA). Leaf samples for biochemical analyses were collected at 42 and 70 DAA, immediately frozen in liquid nitrogen, and stored under appropriate conditions until analysis. Samples for anatomical evaluation were collected at the end of the experiment (70 DAA).

### 2.2 Plant physiology assessment

#### 2.2.1 Leaf gas exchange

Leaf gas-exchange measurements were performed on six leaves per treatment, during the morning period (9:00 - 11:00 AM), at 20, 42, and 70 DAA, using a portable InfraRed Gas Analyzer (LCpro+, ADC BioScentific Ltd., Hoddesdon, UK) equipped with a 6.25 cm^2^ leaf chamber. Net CO_2_ assimilation rate (A, µmol·m^−2^ s^−1^), stomatal conductance (gs, mmol·m^−2^ s^−1^), transpiration rate (E, mmol·m^−2^ s^−1^), and ratio of intercellular to atmospheric CO_2_ concentration (Ci/Ca) were calculated using the equations of von Caemmerer and Farquhar, 1981. The A/gs (µmol·mol^−1^) ratio was used to estimate the intrinsic water use efficiency.

#### 2.2.2 Chlorophyll *a* fluorescence

Chlorophyll *a* fluorescence parameters were measured at 20, 42 and 70 DAA on the same leaves used for gas exchange measurements, using a Field Portable Pulse Modulated Chlorophyll Fluorescence Measuring System Complete (FMS2+; Hansatech Instruments; Walz, King’s Lynn, England). Measurements were conducted during the morning period (09:00 -11:00 AM), using two scripts.

In the first script, measurements were performed on leaves that were fully exposed to sunlight. For this procedure after a 15 s exposure to actinic light (1500 μmol m^−2^ s^−1^), light-adapted steady-state fluorescence yield (Fs) was averaged, followed by exposure to a saturating light pulse (5100 μmol m^−2^ s^−1^) for 0.7 s to establish Fm’. The sample was then shaded for 11 s with a far-red light source to determine F0’. In the second script, using the dark leaf clip, the same leaf portion used in the first script was immediately dark acclimated for 30 min. After this, the maximum photochemical efficiency of PSII was given by Fv/Fm = (Fm - F0)/Fm, where F0 corresponds to the minimum fluorescence level excited by the very low intensity of the measuring light to keep PSII reaction centers open, and Fm corresponds to the maximum fluorescence level elicited by a pulse of saturating light (6000 μmol m^−2^ s^−1^) which closes all PSII reaction centers (Bilger and Schreiber, 1986).

According to these measurements, the following fluorescence attributes were calculated: effective quantum efficiency of photosystem II (ΦPSII), photochemical fluorescence quenching (qP) and non-photochemical quenching (NPQ) and apparent electron transport rate (ETR).

#### 2.2.3 Chlorophyll *a* fluorescence transient analysis using the JIP-test

The chlorophyll transients of dark-acclimated (30 min) attached grapevine leaves were also measured during the morning period (09:00-11:00 AM). For this, a portable Handy-PEA^®^ chlorophyll fluorometer (Handy-Plant Efficiency Analyser, Hansatech Instruments, King’s Lynn, Norfolk, UK) was used according to Brito et al., 2024. The transients were induced by 1 s illumination with an array of 3 light-emitting diodes with a maximum light intensity of 3000 µmol m^–2^ s^–1^ and a homogeneous irradiation over a 4 mm diameter leaf area. The fast fluorescence kinetics (F_0_ to F_m_) was recorded from 10 µs to 1 s. The fluorescence intensity at 50 µs was considered F_0_ (Strasser et al., 2000).

The translation of the measured parameters into JIP-test parameters provided structural and functional information and allowed to quantify the PSII behavior of different varieties. The biophysical parameters derived from the OJIP transients were calculated according to the JIP-test equations (Strasser and Tsimilli-Michael, 2001; Strasser et al., 2010; Stirbet and Govindjee, 2011) and following the details described in Dinis et al., 2016. Briefly, the following variables were determined: the normalized area above the OJIP transient (reflecting the number of reduction and oxidation of one Q_A_^-^ molecule during the fast OJIP transient and therefore related to the electron carriers per electron transport chain) (S_M_); the average absorbed photon flux per PSII RC (ABS/RC); the maximum trapped excitation flux at time zero per PSII (leading to QA reduction) (TR_0_/RC); the relative variable fluorescence at 30 ms (V_I_); the dissipated energy flux at time zero per PSII (DI_0_/RC); the electron transport flux at time zero per PSII (ET_0_/RC); the probability with which a PSII trapped exciton moves an electron into the electron transport chain beyond Q_A_^-^ (Ψ_0_), the quantum yield of the electron transport flux beyond Q_A_^-^ (φE_0_) and the performance index (PI_ABS_).

### 2.3 Biochemical responses assessment

#### 2.3.1 Quantification of phenolic content and antioxidant activity

To determine the secondary metabolites and antioxidant capacity in the leaves, a methanolic extract at a concentration of 4 mg mL^-1^ was prepared and used for the following quantifications.

Total phenols were quantified using the Folin-Ciocalteu method, with absorbance measured at 725 nm, as described by Rodrigues et al., 2015. Quantification was performed using a gallic acid standard curve in the range of 0.0078–2 mg mL^-1^, with a coefficient of determination of *R^2^* = 0.9998. Results were expressed as milligrams per gram of fresh weight (mg GAE g^-1^ FW). Flavonoid content in the extracts was quantified using the aluminum chloride (AlCl_3_) complex method, with absorbance measured at 510 nm (Rodrigues et al., 2015). Quantification was performed using a catechin standard curve in the range of 0.00039–1 mg mL-1, with a coefficient of determination of *R^2^* = 0.9995. Results were expressed as milligrams of catechin equivalents per gram of FW (mg CAE g^-1^ FW). *Ortho*-diphenols were measured by reading the absorbance ate 370 nm. A gallic acid standard curve ranging from 0.0078 to 2 mg mL^-^¹, with a coefficient of determination *R^2^* = 0.9927, was used to quantify these compounds. Results were expressed in milligrams of gallic acid equivalents per gram of FW (mg GAE g^-1^ FW) (Baltazar et al., 2025b). The radical scavenging capacity using ABTS (2,2′-azino-bis (3-ethylbenzothiazoline-6-sulphonic acid)) assay was performed according to Baltazar et al., 2025b. The ABTS•+ working solution was prepared by reacting 7 mol m^-3^ ABTS with 140 mol m^-3^ K_2_S_2_O_8_ in ultra-pure water, followed by incubation in the dark at room temperature for 12–16 hours. After that, the solution´s absorbance was adjusted to 0.7-0.8 at 734 nm. Sample absorbance and calibration curve readings were recorded at 734 nm. Quantification was performed using a Trolox standard curve in the range of 0.00195 - 0.5 mmol mL^-1^ with a coefficient of determination *R^2^* = 0.9927. Results regarding the ABTS•+ scavenging activity were expressed as mmol TE g^-1^ FW. Percent inhibition and TEAC were calculated based on absorbance readings, comparing sample absorbance to controls and blanks, following the equations:


% inhibition=100×(Abs734 blank−Abs734 sample)/Abs734 blank

TEAC(mmol TE g−1 FW)=(% inhibition−b)/a,

where a is the slope of the standard curve (y = ax + b); b is the y-intercept.

#### 2.3.2 Photosynthetic pigments

To quantify the photosynthetic pigments, 4 mL of 80% acetone were added to 10 mg of fresh leaf tissue. The samples were homogenized using a vortex mixer followed by ultrasonication for 5 min. The resulting extracts were then centrifuged at 1610 RCF for 10 min at 4 °C. Absorbance reading was taken at 663 nm, 645 nm, and 479 nm using a SPECTRUM star Nano spectrophotometer (BMG Labtech GmbH, Germany). Given the light and temperature sensitivity of these pigments, all extraction and quantification steps were performed on ice and shielded from light. Pigment concentrations were calculated according to Arnon (1949) and Lichtenthaler (1987), with results expressed in mg g^-1^ FW.

#### 2.3.3 Soluble sugars

To quantify soluble sugars, 10 mg of each sample were extracted with 5 mL of 80% (v/v) ethanol. The extracts were homogenized and incubated in a water bath at 80 °C for 60 min, after which the supernatant was collected. An aliquot of the extract was then mixed with anthrone reagent, vortexed thoroughly, and heated at 100 °C for 10 min in a water bath. After cooling, absorbance was measured at 625 nm (Leyva et al., 2008). A glucose standard curve in the range of 1 - 100 µg mL^-1^, with a coefficient of determination *R^2^* = 0.9966, was used for quantification. Results were expressed as mg g^-1^ FW.

#### 2.3.4 Soluble proteins

Soluble proteins were extracted using a phosphate-based extraction buffer (pH 7.5), supplemented with EDTA (ethylenediaminetetraacetic acid). The working solution contained this extraction buffer along with PMFS (polyvinylpyrrolidone). Protein quantification followed the Bradford method (Bradford, 1976), with absorbance measured at 595 nm. Quantification was performed using a bovine serum albumin (BSA) standard curve in the range of 0.0039–1 mg mL^-1^, with a coefficient of determination *R^2^* = 0.999. Soluble protein content was expressed as milligrams of BSA equivalents per gram of fresh weight (mg BSAE g^-1^ FW).

#### 2.3.5 Proline

To determine proline, 200 mg of each sample was extracted in 1 mL of 3% SSA (sulfosalicylic acid), followed Bernardo et al., 2022. The extracts were homogenized in 3 cycles of ultrasound (15 s), ice and vortex, and after centrifuged at 3500 rpm for 20 minutes at 4 °C. Next, to each supernatant sample was added glacial acetic acid and ninhydrin and heated at 100 °C in a water bath for 60 minutes. After waiting until it cools down on ice and added 1 mL of toluene and mixed on vortex. Absorbance was measured at 520 nm. A proline standard curve in the range of 0.025 -1 µmol mL^-1^, with a coefficient of determination *R^2^* = 0.9985, was used for quantification. Results were expressed as mg g^-1^ FW. All absorbance readings were taken using a SPECTRUM star Nano spectrophotometer (BMG Labtech GmbH, Germany).

### 2.4 Leaf anatomical traits

For leaf histological analysis, four healthy and fully expanded leaves per treatment (n = 4) were collected. Small leaf segments from the median region of the leaf blade were fixed in FAA solution (formaldehyde, alcohol, and glacial acetic acid; 90:5:5, v/v). The samples were processed according to routine histological procedures, with paraffin embedding (Johansen, 1940). Transverse sections of 6 µm thickness were made using a rotary microtome (Leica RM2016, Germany), stained with 0.1% toluidine blue. The sections were observed and photographed using a specific optical microscope (Olympus IX51, Olympus Corporation, Tokyo, Japan), and morphometric analysis was performed using Digimizer^®^ software (version 6.5.0, MedCalc Software’s, Ostend, Belgium).

### 2.5 Statistical analysis

Statistical analysis was performed using the IBM SPSS^®^ Statistics program (v. 26). After testing for ANOVA assumptions (homogeneity of variances with the Levene’s mean test, and normality with the Kolmogorov-Smirnov test), statistical differences between treatments within each timepoint were evaluated by one-way and two-way factorial ANOVA. Means were separated with the Tukey’s *post-hoc* test at 5% level.

## 3 Results and discussion

### 3.1 Climatic conditions and leaf temperature

The **Figure 1** shows the air temperature and precipitation values recorded at the study site. From late June onwards, maximum temperatures frequently exceeded 35 °C, with several episodes exceeding 40 °C, indicating the occurrence of heatwaves. During July, daily maximum temperatures remained 34-38 °C. August emerged as the most thermally stressful month, with repeated days ≥ 40 °C and periods above 35 °C. In September, maximum temperatures above 30-35 °C persisted during the first half of the month.

**Figure 3:**
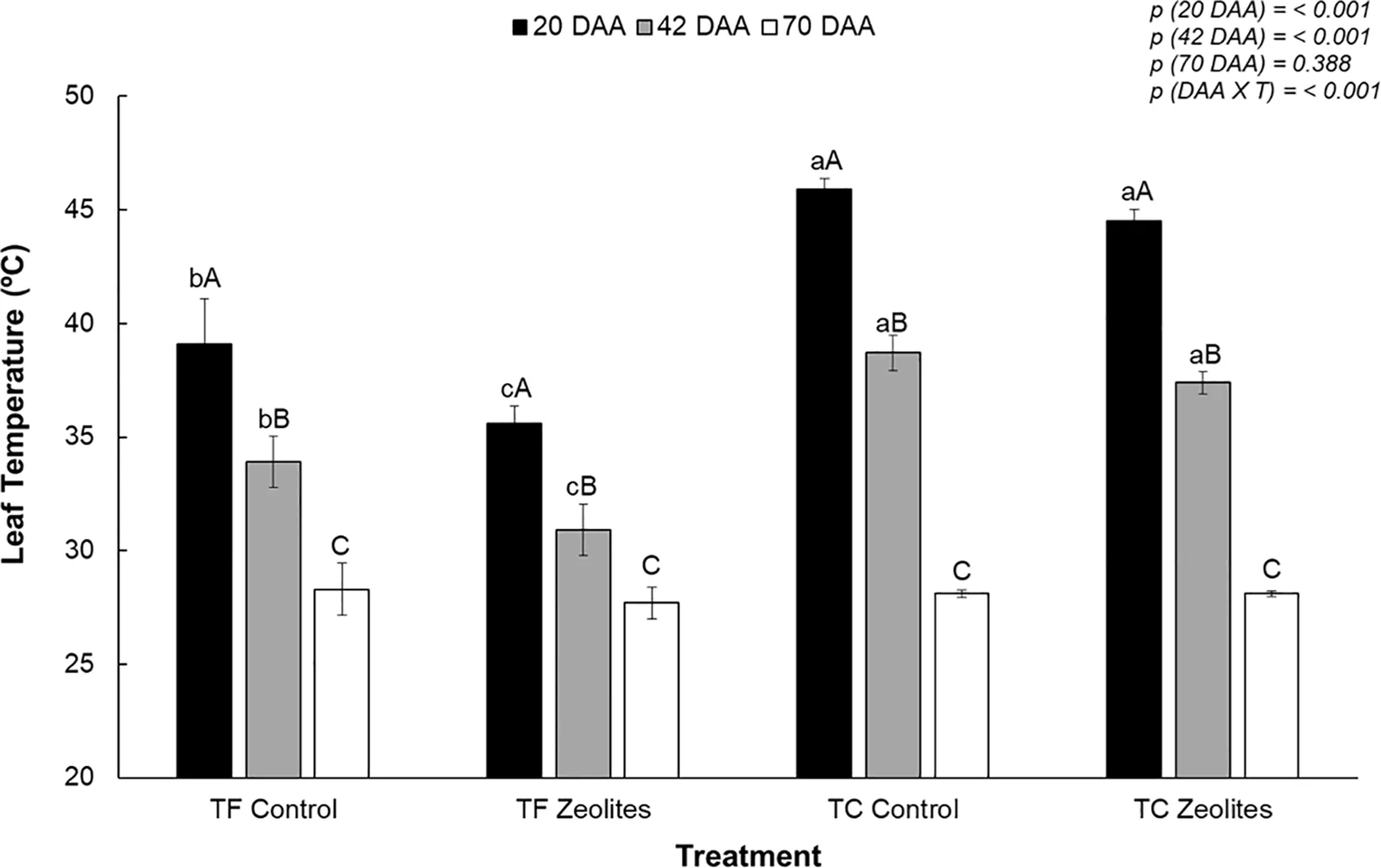
Evolution of leaf temperature in TF, Touriga Franca and TC, Tinto Cão varieties, treated with zeolites (TF Zeolites; TC Zeolites) and untreated - control (TF Control; TC Control), 20, 42 and 70 DAA, days after application. Each point with vertical bars represents the average and SD, respectively. Different lowercase letters indicate significant differences between treatments, while uppercase letters denote significant differences between timepoints.

Regarding leaf temperature (**Figure 3**), zeolite application significantly mitigated heat stress throughout the 3 timepoints, as reflected by the consistently lower leaf temperatures from 20 to 70 DAA in both varieties and treatments. This represents one of the most consistent response observed in the present study, highlighting the direct impact of zeolite application on canopy thermal regulation. At 20 and 42 DAA, zeolite significantly reduced leaf temperature in TF variety (*F*_3,17_ = 49.590; *p* = 1.28^-08^), by 3.5 °C, and 3.0 °C respectively, whereas no statistically significant differences were observed at 70 DAA. In TC, although differences were not statistically significant, a consistent cooling trend was observed, with reductions of 1.4 °C at 20 DAA and 1.3 °C at 42 DAA. Notably, these reductions in leaf temperature reflect a clear alleviation of thermal load at the leaf level under field conditions. Thus, foliar application of zeolites proved to be an effective short-term strategy for mitigating canopy thermal load under the climatic conditions of the DDR. This cooling effect is commonly attributed to the optical properties of the particle films, namely the small diameter of the particles, which increase leaf reflectance and reduce the absorption of infrared radiation, thereby decreasing leaf energy balance and thermal stress (Cataldo et al., 2021; Conversa et al., 2024). Notably, TF showed a more pronounced thermal response to zeolite application than TC, suggesting that variety-specific traits, such as canopy architecture, leaf orientation, or intrinsic heat sensitivity, modulate the effectiveness of these films. Similar cooling effects in Mediterranean vineyards have been recently documented, reinforcing zeolites as a robust tool against extreme heat (Petoumenou, 2023; Cataldo et al., 2025).

### 3.2 Distinct physiological adjustments in different varieties

Chlorophyll *a* fluorescence analysis, including OJIP transients, together with leaf gas exchange measurements, constitute powerful and complementary tools for assessing plant physiological performance under contrasting treatments and environmental conditions. These tools allow the evaluation of photosynthetic process at multiple levels, from stomatal regulation and carbon assimilation to excitation energy absorption and photochemical efficiency in photosystem II. When complemented by targeted biochemical analyses, this integrated framework enables to evaluate varietal differences in stress tolerance, to assess the effectiveness of mitigation strategies, and to elucidate variety-specific physiological responses to summer stress conditions.

#### 3.2.1 Photochemical parameters, stomatal and photosynthetic traits

Significant differences between treatments were detected for several chlorophyll *a* fluorescence parameters, with significant interactions, between measurement date and treatment (DAA × T), observed for F_0_ (*F*_6_ = 5.690; *p* = 0.0001), ΦPSII (*F*_6_ = 9.049; *p* = 1.70^-06^), qP (*F*_6_ = 6.727; *p* = 0.00004), ETR (*F*_6_ = 5.668; *p* = 0.0002), and NPQ (*F*_6_ = 8.620; *p* = 2.96^-06^) (**Table 2**).

**Table 2:** Evolution of chlorophyll a parameters in TF, Touriga Franca and TC, Tinto Cão varieties, treated with zeolites (TF Zeolites; TC Zeolites) and untreated - control (TF Control; TC Control), 20, 42 and 72 DAA, days after application.

DAA	Treatment	F_0_		F_v_/F_m_		Φ_PSII_		qP		ETR		NPQ	
**20**	TF Control	220.8 ± 25.8	bB	0.698 ± 0.076	B	0.195 ± 0.042	ab	286.8 ± 100.8	B	22.0 ± 15.9	B	0.626 ± 0.416	B
	TF Zeolites	189.2 ± 27.7	bB	0.746 ± 0.039	AB	0.226 ± 0.023	aA	307.0 ± 38.4	B	29.7 ± 2.79	B	0.560 ± 0.225	B
	TC Control	201.8 ± 12.1	bB	0.785 ± 0.023		0.166 ± 0.039	abA	223.6 ± 28.0	B	21.6 ± 13.7	B	0.985 ± 0.526	B
	TC Zeolites	312.8 ± 55.7	aB	0.711 ± 0.083	AB	0.170 ± 0.015	b	296.1 ± 49.3	B	24.6 ± 5.61		0.848 ± 0.747	B
	***p value***	<0.001		n.s.		0.026		n.s.		n.s.		n.s.	
**42**	TF Control	225.5 ± 6.61	B	0.801 ± 0.008	A	0.165 ± 0.016	bc	0.387 ± 0.048	abB	31.1 ± 3.09	bAB	0.740 ± 0.061	aB
	TF Zeolites	217.8 ± 19.4	B	0.798 ± 0.024	A	0.239 ± 0.022	aA	0.478 ± 0.054	aA	45.1 ± 4.12	aA	0.507 ± 0.143	abB
	TC Control	234.6 ± 19.9	B	0.794 ± 0.025		0.192 ± 0.026	bA	0.353 ± 0.012	bA	33.2 ± 4.58	bA	0.394 ± 0.176	bB
	TC Zeolites	208.2 ± 28.8	C	0.766 ± 0.025	A	0.146 ± 0.023	c	0.300 ± 0.074	bB	27.9 ± 4.46	b	0.366 ± 0.145	bB
	***p value***	n.s.		n.s.		<0.001		<0.001		<0.001		0.006	
**70**	TF Control	577.5 ± 13.5	A	0.735 ± 0.028	AB	0.196 ± 0.019	a	0.466 ± 0.025	aA	37.0 ± 3.63	aA	2.67 ± 0.378	bA
	TF Zeolites	624.0 ± 65.3	A	0.725 ± 0.056	B	0.153 ± 0.030	aB	0.373 ± 0.045	aB	23.1 ± 2.69	bB	3.45 ± 0.238	aA
	TC Control	629.4 ± 28.1	A	0.732 ± 0.027		0.098 ± 0.012	bB	0.199 ± 0.024	bB	17.9 ± 1.64	bB	2.62 ± 0.415	bcA
	TC Zeolites	651.8 ± 37.1	A	0.693 ± 0.028	B	0.180 ± 0.030	a	0.383 ± 0.090	aA	34.0 ± 5.63	a	1.92 ± 0.489	cA
	***p value***	n.s.		n.s.		<0.001		<0.001		<0.001		<0.001	
***p value (DAA X T)***		<0.001		n.s.		<0.001		<0.001		<0.001		<0.001	

Values are presented as means ± SD. The results show differences between treatments and the interaction between treatment and DAA. Statistical significance was determined using Tukey’s HSD test at p < 0.05. Different lowercase letters indicate significant differences between treatments, while uppercase letters denote significant differences between timepoints. “n.s.” indicates non-significant differences between treatment.Minimum fluorescence yield (F0), Maximum (Fv/Fm) and effective (ΦPSII) quantum efficiency of photosystem II; qP, photochemical and NPQ, non-photochemical fluorescence quenching and apparent ETR, electron transport rate.

As expected, seasonal changes in meteorological conditions (**Figure 1**) led to marked temporal variability in most parameters in both TF and TC varieties, under both control and zeolite treatments. At 20 DAA, zeolite-treated TC plants exhibited higher F_0_ (*F*_3,15_ = 12.973; *p* = 0.0002) and lower ΦPSII (*F*_3,15_ = 4.080; *p* = 0.026) compared with zeolite-treated TF. The increase in F_0_ suggests a higher proportion of inactive or damaged PSII reaction centers (Baker, 2008), likely reflecting a transient acclimation to the reduced light availability (shading effect) induced by the film. At the same time, the decrease in ΦPSII reflects limitations in effective photochemical energy conversion (Baker, 2008). Given the naturally low light saturation point and conservative light-harvesting strategy of TC observed by Brito et al., 2024, this variety appears more sensitive to initial alterations in leaf optical properties. Conversely, at 42 DAA, zeolite significantly enhanced ΦPSII (*F*_3,15_ = 15,775; *p* = 0.00007) and ETR (*F*_3,15_ = 16.162; *p* = 0.00006) in TF plants, indicating improved photochemical efficiency and electron transport efficiency through photosystem II during peak summer stress (Baker, 2008). Similar enhancements in PSII performance following zeolite particle film application have been reported in grapevines exposed to high irradiance and temperature, especially under stress (Valentini et al., 2021; Cataldo et al., 2025).

In parallel, the higher qP (*F*_3,15_ = 10.245; *p* = 0.001) and NPQ (*F*_3,15_ = 6.200; *p* = 0.006) values observed in both TF treatments (control and zeolite) compared with TC suggest a greater fraction of open PSII reaction centers and a superior capacity for dissipate excess excitation energy, respectively (Baker, 2008). These responses highlight the high photosynthetic plasticity of TF under the prevailing environmental conditions. The reduction in ΦPSII and qP in treated TC plants suggests a variety-specific sensitivity, in which the particle film may limit light harvesting during mid-summer. In contrast, the relatively stable NPQ observed in TC across treatments supports the notion of a constitutive photoprotective strategy based on sustained thermal energy dissipation, as previously reported for this variety (Brito et al., 2024). At 70 DAA, TC control plants exhibited reduced qP (*F*_3,15_ = 19.700; *p* = 0.00001) and ΦPSII (*F*_3,15_ = 14.949; *p* = 0.0001), indicative of a decline in PSII efficiency at later stages, while zeolite-treated TC plants exhibited higher photochemical efficiency (superior ΦPSII, qP, and ETR), reaching values comparable to TF plants, and reducing their reliance on NPQ (lower) (*F*_3,15_ = 12.716; *p* = 0.0002), which points to a strong long-term acclimation capacity to zeolites.

**Figure 4:**
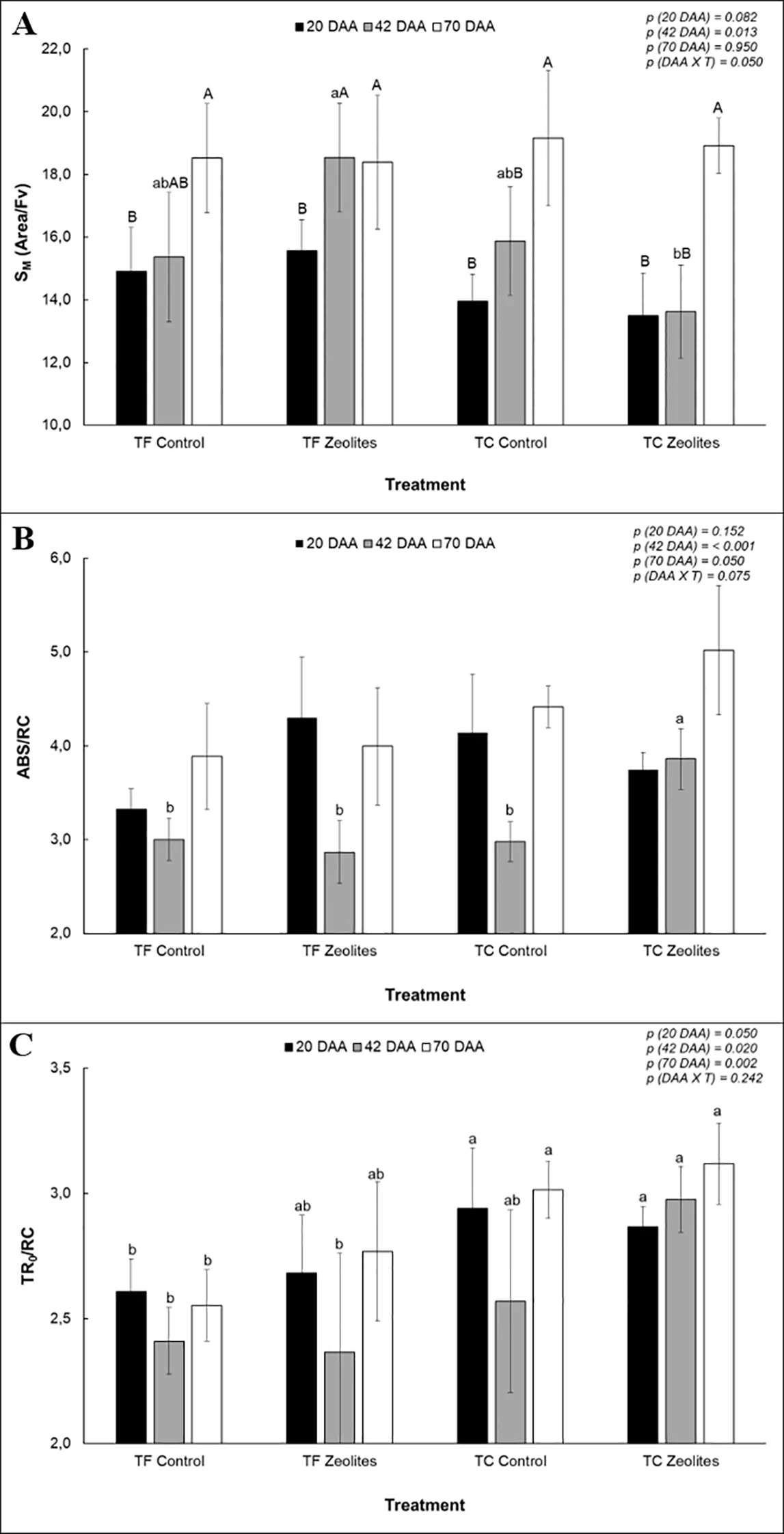
Evolution of transient JIP test parameters in TF, Touriga Franca and TC, Tinto Cão varieties, treated with zeolites (TF Zeolites; TC Zeolites) and untreated - control (TF Control; TC Control), 20, 42 and 70 DAA, days after application. Normalized area above the OJIP transient **(A)**, Average absorbed photon flux per PSII RC **(B)**, Maximum trapped excitation flux at time zero per PSII **(C)**. Each point with vertical bars represents the average and SD, respectively. Different lowercase letters indicate significant differences between treatments, while uppercase letters denote significant differences between timepoints, according Tukey’s HSD test at *p* < 0.05. The absence of letters indicates no significant differences among treatments and/or timepoints.

The S_M_ parameter was significantly higher (*F*_2,43_ = 29.444; *p* = 8.81^-09^) in zeolite-treated TF at 42 DAA (**Figure 4A**), suggesting a larger electron acceptor pool or greater capacity to reduce electron acceptors beyond Q_A_^-^ (Strasser et al., 2010). The opposite was observed for TC, in which zeolite treatment led to a decrease of S_M_ at 42 DAA. Nonetheless, at 70 DAA no significant differences were observed between treatments. In fact, across all treatments, S_M_ increased significantly with time, reaching the highest values at 70 DAA, suggesting an overall enhancement at later stages of development.

By contrast, ABS/RC and TR_0_/RC remained relatively stable throughout the experimental period (**Figures 4B, C**, respectively), indicating relative stability in the apparent antenna size and in the trapping flux per PSII reaction center (Dąbrowski et al., 2024). A slight increase in ABS/RC (*F*_2,43_ = 29.184; *p* = 9.83^-09^) and TR_0_/RC (*F*_2,43_ = 7.583; *p* = 0.002) was observed in zeolite-treated TC plants (with similar value to TC control) at 42 and 70 DAA, which could reflect a compensatory enlargement of the functional antenna size to offset reduced light absorption. This response may also be associated with a lower density of active reaction centers, which become non-Q_A_-reducing and act as “heat dissipators” to protect PSII (Strasser et al., 2004; Tsimilli-Michael, 2019). This interpretation is aligned with the significant increase in dissipation energy per reaction center (DI_0_/RC) (*F*_2,43_ = 58.618; *p* = 5.21^-13^) observed at these timepoints, particularly in the TC variety, as discussed below.

**Figure 5:**
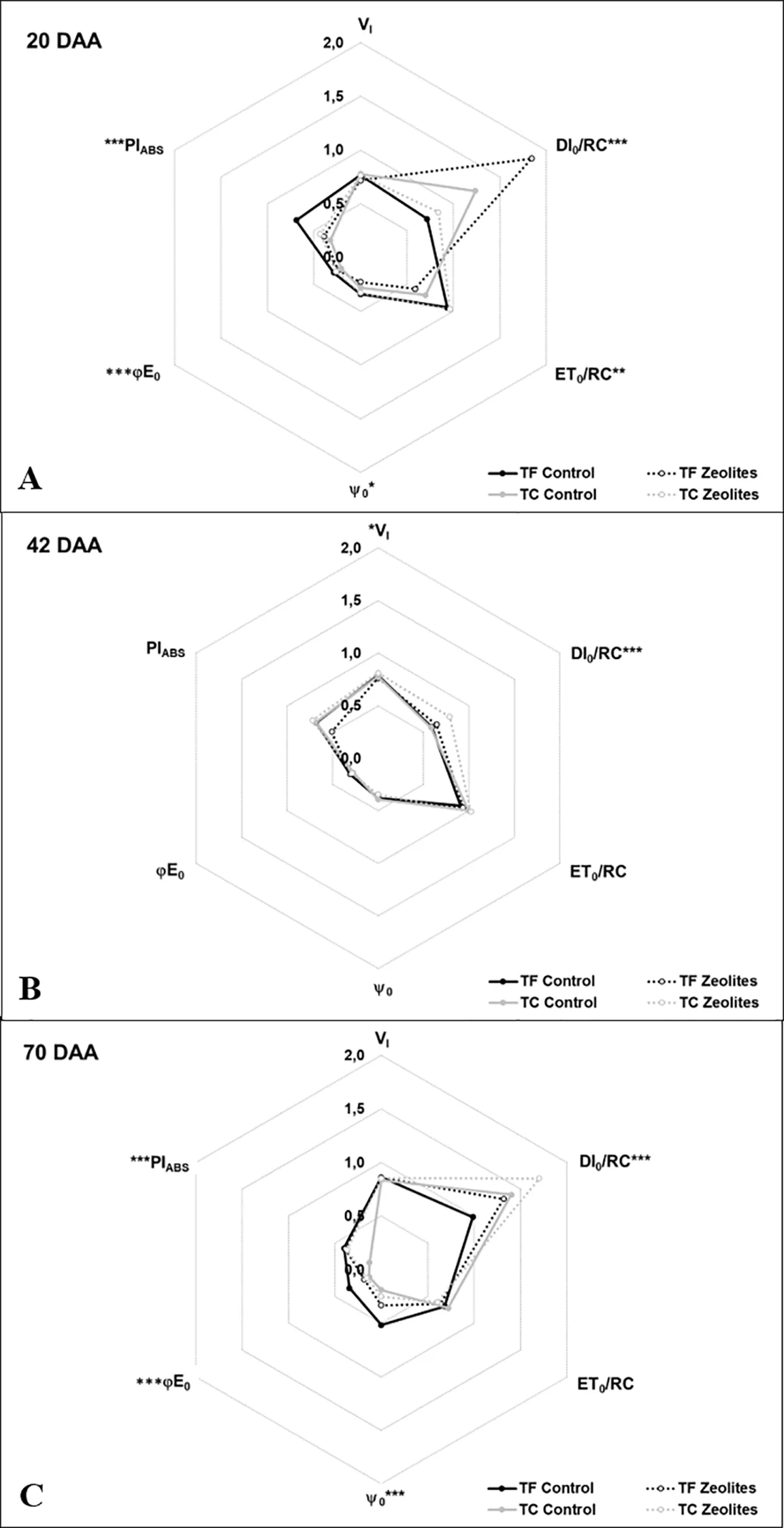
Spider plot of JIP parameters deduced from chlorophyll a fluorescence OJIP transient curves in zeolite-treated and control Touriga Franca (TF) and Tinto Cão (TC) varieties, 20 **(A)**, 42 **(B)** and 70 **(C)** days after application (DAA). Relative variable fluorescence at 30 ms (V_I_); Dissipated energy flux at time zero per PSII (DI_0_/RC); Electron transport flux at time zero per PSII (ET_0_/RC); the efficiency/probability with which a PSII trapped electron is transferred beyond Q_A_^-^ (Ψ_0_), the quantum yield of the electron transport flux beyond Q_A_^-^ (φE_0_); and the performance index (PI_ABS_). Statistical significance was determined using Tukey’s HSD test at *p* < 0.05. *** *p*<0.001, ** *p*<0.01.

**Figure 5** presents a multiparameter radar plot of the parameters deduced from chlorophyll *a* fluorescence OJIP transient curves. Significant DAA × T interactions were detected for DI_0_/RC (*F*_6,43_ = 17.187; *p* = 5.17^-10^), ψ_0_ (*F*_6,43_ = 10.496; *p* = 3.75^-07^), φE_0_ (*F*_6,43_ = 4.795; *p* = 0.001), and PI_ABS_ (*F*_6,43_ = 5.123; *p* = 0.0005), highlighting the dynamic and variety-dependent modulation of PSII energy fluxes in response to both seasonal progression and zeolite application. However, as shown in **Figure 5**, the differences across treatments are most obvious in the initial (20 DAA) and final (70 DAA) phases of the experiment.

At control level, clear variety differences were evident. At 20 DAA (**Figure 5A**), TF control plants displayed a higher ψ_0_ (*F*_2,43_ = 8.651; *p* = 0.001), φE_0_ (*F*_2,43_ = 19.305; *p* = 1.04^-06^), ET_0_/RC (*F*_2,43_ = 18.957; *p* = 1.25^-06^) and DI_0_/RC (*F*_2,43_ = 58.618; *p* = 5.21^-13^) relative to TC control plants (Tsimilli-Michael, 2019; Horazeck et al., 2020; Dąbrowski et al., 2024). These baseline observations are in accordance with previous reports characterizing TC as having lower PSII photochemical efficiency and higher energy dissipation compared to other varieties (Brito et al., 2024). Consequently, the PI_ABS_ (*F*_2,43_ = 42.101; *p* = 7.46^-11^), reflects a superior energy conservation from photons absorbed by PSII until the reduction of intersystem electron acceptors (Strasser et al., 2010; Dąbrowski et al., 2024) reached its highest value in TF control at 20 DAA. Indeed, intraspecific variations between varieties regarding PSII functionality and specific stress responses are observed (Xu et al., 2014; Jedmowski and Brüggemann, 2015; Brito et al., 2024), which likely dictate different shifts in energy dissipation and overall performance index observed in response to foliar zeolite application as observed in this study. For instance, at the early stage (20 DAA), zeolites acted as a protective buffer for TC, decreasing DI_0_/RC relative to its control and improving most electron transport parameters (ψ_0_, φE_0_ and ET_0_/RC). This is also corroborated by the slightly higher ETR and lower NPQ denoted in **Table 2**. In contrast, for TF, the application of zeolites led to a substantial increase in DI_0_/RC relative to its control, marking an early shift toward photoprotection while decreasing energy flux in the acceptor side of PSII (ψ_0_, φE_0_ and ET_0_/RC) (Tsimilli-Michael, 2019; Horazeck et al., 2020).

Nonetheless, these differences were largely attenuated by 42 DAA, with most parameters becoming similar across treatments, suggesting initial differences could arise from a period of acclimation (**Figure 5B**). Importantly, as 42 DAA coincide with exposure of the plants to peak-summer stress conditions, the absence of negative effects on these PSII photochemistry parameters under these conditions show that foliar zeolite application do not cause harm to the plant under severe stress, which is in accordance with previous reports (Petoumenou, 2023; Valentini et al., 2025).

At the later stage (70 DAA), the basal variety differences observed at 20 DAA remained visible, with control TF maintaining a higher PI_ABS_ (*F*_2,43_ = 9.111; *p* = 0.0001) than control TC (**Figure 5C**). However, the zeolite treatment led the PI_ABS_ of TC to match that of TF (both control and zeolite treated), indicating a beneficial late-season stabilization for TC. At this stage, zeolite treatment led to increased energy dissipation in both varieties and caused a reduction in φE_0_ (*F*_2,43_ = 2.838; *p* = 0.049) and ψ_0_ (*F*_2,43_ = 32.285; *p* = 4.34^-11^) specifically in TF, which is also reflected in the decrease in ETR (**Table 2**). Under high temperature stress, electron transport beyond Q_A_^-^ is typically decreased (Luo et al., 2011; Sun et al., 2016). These changes can, however, be interpreted as a photoprotective mechanism of downregulating photochemical reactions to withstand heat stress conditions (Brito et al., 2024). This is corroborated by the similar or increased levels of F_v_/F_m_, ΦPSII, and qP of zeolite treated plants relative to the control observed in this study (**Table 2**) and reported in the literature (Petoumenou, 2023; Valentini et al., 2025).

**Figure 6:**
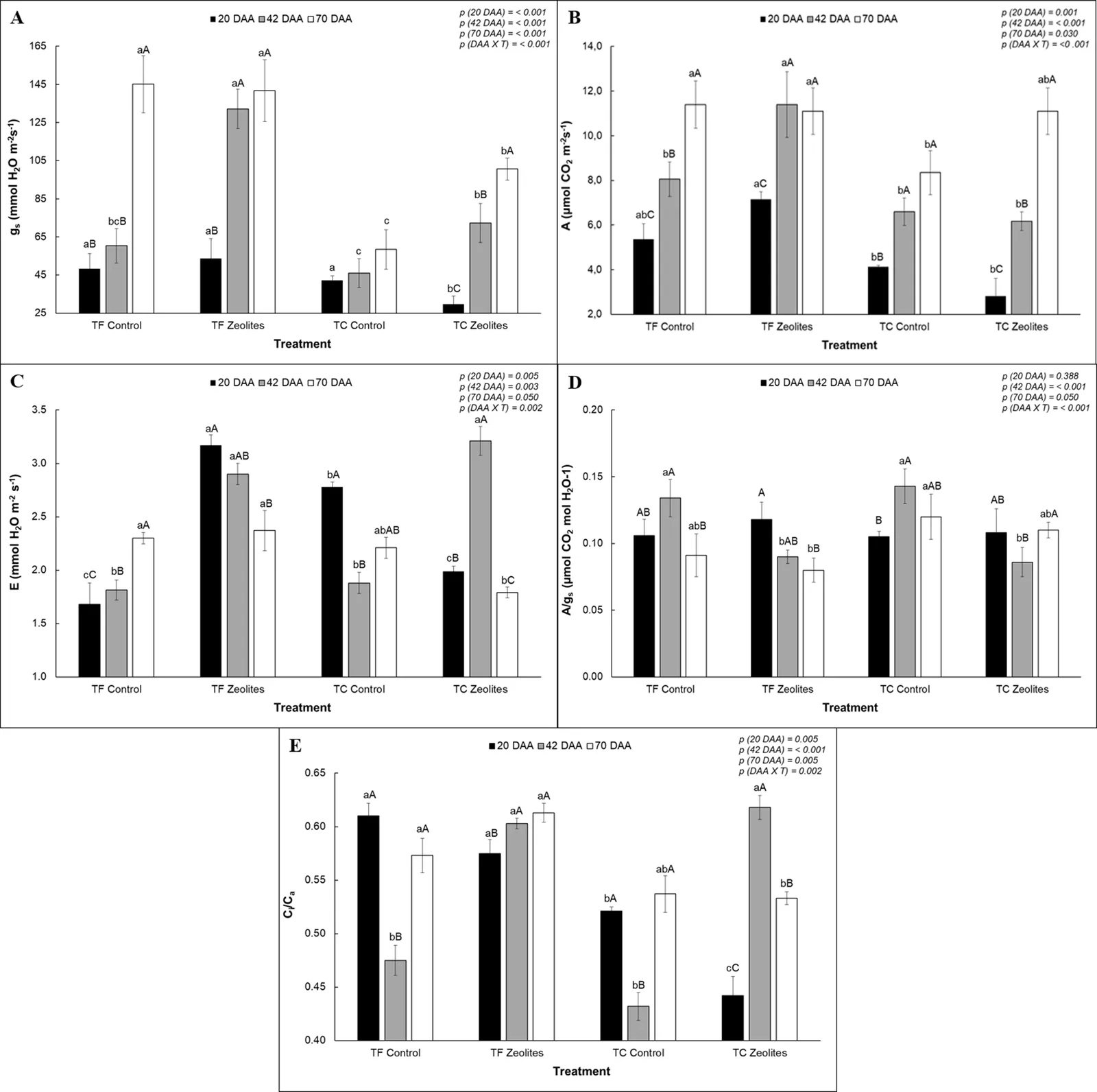
Evolution of leaf gas exchange variables in TF, Touriga Franca and TC, Tinto Cão varieties, treated with zeolites (TF Zeolites; TC Zeolites) and untreated - control (TF Control; TC Control), 20, 42 and 70 DAA, days after application. Net photosynthetic rate **(A)**, stomatal conductance **(B)**, Transpiration rate **(C)**, Intrinsic water use efficiency (A/gs) **(D)** and intercellular to atmospheric CO_2_ concentration (Ci/Ca) **(E)**. Each point with vertical bars represents the average and SD, respectively. Different lowercase letters indicate significant differences between treatments, while uppercase letters denote significant differences between timepoints.

Regarding the leaf gas exchange results presented in **Figure 6**, at 20 DAA, zeolite application in TF variety induced a significant increase in g_s_ (*F*_3,16_ = 23.866; *p* = 3.78^-06^), A (*F*_3,18_ = 8.44; *p* = 0.001) and E (*F*_3,18_ = 5.975; *p* = 0.005). At the same timepoint, TF plants under both control and zeolite treatments exhibited higher Ci/Ca than TC variety, suggesting sustained CO_2_ availability within the leaf mesophyll. This response may be associated with increased g_s_ in TF and reduced photochemical demand in TC assumed by the accumulation with lower g_s_. At 42 DAA, zeolite-treated TF plants maintained this stimulation of g_s_ (*F*_3,15_ = 39.568; *p* = 2.31^-07^)and A (*F*_3,15_ = 20.843; *p* = 0.00001), whereas controls of both varieties shifted toward a more conservative strategy (higher A/g_s_) (*F*_3,15_ = 16.626; *p* = 0.00005).

By 70 DAA, TC control plants displayed the lowest g_s_ (*F*_3,15_ = 12.516; *p* = 0.0003) and A values (*F*_3,15_ = 3.915; *p* = 0.030), indicating strong late season limitations, while, zeolite-treated TC plants showed the lowest E and C_i_/C_a_ ratio, suggesting reduced transpirational water loss and tighter stomatal control. Consistently, this variety exhibited the highest A/g_s_, pointing to a strategy favoring water conservation over maximal carbon assimilation under prolonged seasonal stress.

Amelioration of water status, increases water use efficiency, along with improvements and stomatal conductance and net photosynthetic rates are also usually reported with zeolites foliar application, enhancing CO_2_ concentration at stomatal level while enable a better water conservation (De Smedt et al., 2017; Petoumenou, 2023; Cataldo et al., 2025; Valentini et al., 2025). The coordinated enhancement of g_s_, A, ΦPSII and ETR, demonstrates a synergy between photochemical and diffusional components of photosynthesis. Similar coupling between improved PSII efficiency and increased stomatal conductance under zeolite particle film application has been previously reported in grapevine under stress conditions (Cataldo et al., 2025; Valentini et al., 2025). Zeolite application protected the photosynthetic apparatus through variety-specific pathways, overall, TF benefited from optimized gas exchange and robust electron transport, while TC leveraged the treatment to enhance its intrinsic water-saving strategy through tighter stomatal regulation and long-term photochemical resilience.

### 3.3 Fine-tuning in biochemical parameters

Biochemical responses revealed a fine-tuning of metabolic investment driven by both variety and foliar zeolite application, reflecting distinct strategies of stress mitigation and resource allocation throughout the season.

#### 3.3.1 Phenolic compounds and antioxidant capacity

**Figure 7:**
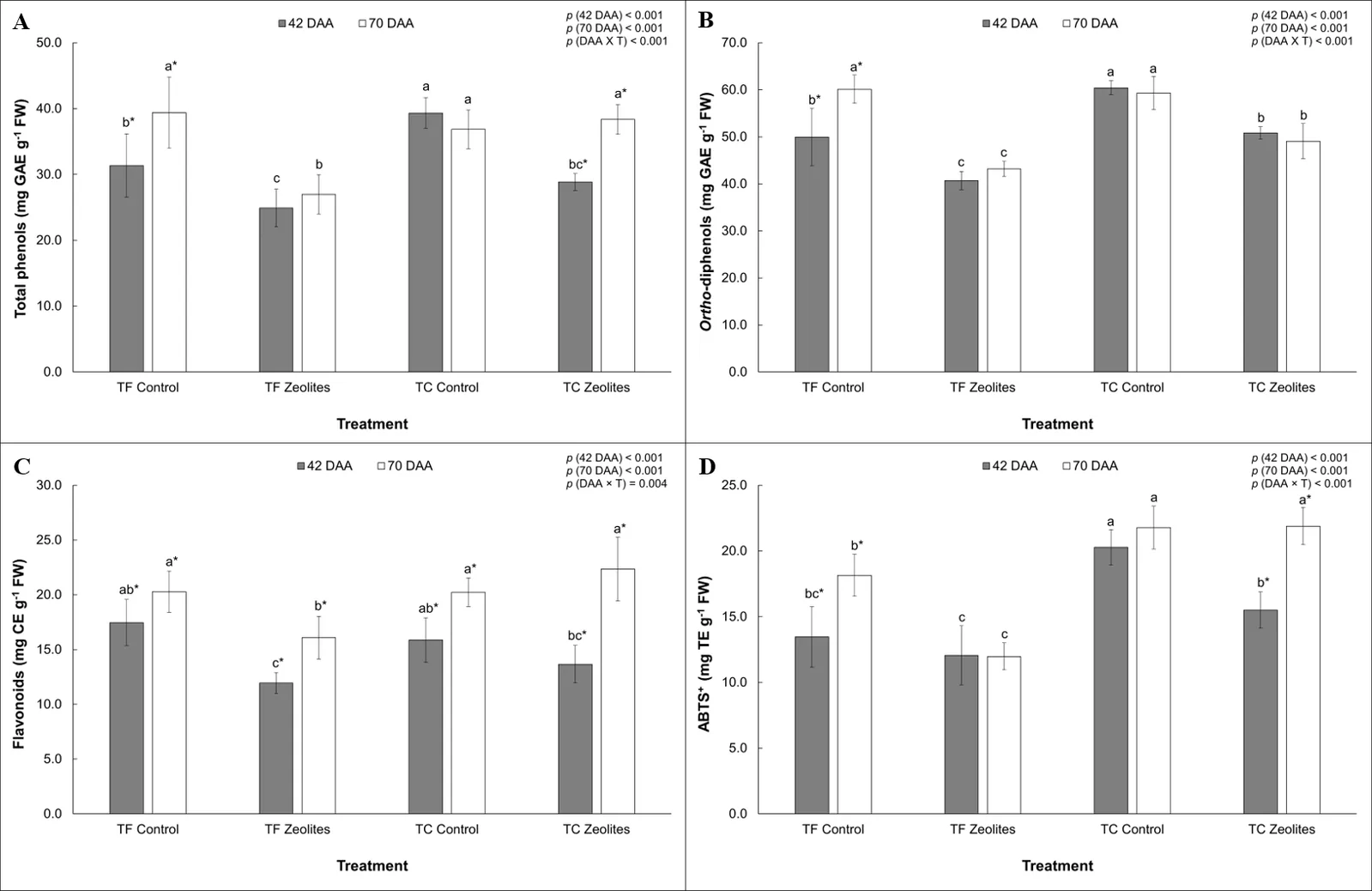
Total phenols **(A)**, ortho-diphenols **(B)**, flavonoids **(C)** and ABTS+ radical scavenging activity **(D)**, measured at 42 and 70 DAA in zeolite-treated and untreated - control TF, Touriga Franca and TC, Tinto Cão varieties. Each point with vertical bars represents the average and SD, respectively. Different letters indicate significant differences between treatments, while * denote significant differences between timepoints, according Tukey’s HSD test at p < 0.05. The absence of letters and/or * indicates no significant differences among treatments and/or timepoints.

**Figure 7** present the results for total phenols, *ortho*-diphenols, flavonoids, and antioxidant capacity (ABTS+). At 42 DAA, total phenols (*F*_3,20_ = 23.056; *p* = 1.06^-06^), *ortho*-diphenols (*F*_3,20_ = 34.717; *p* = 4.02^-08^) and flavonoids (*F*_3,20_ = 11.389; *p* = 0.0001) were all significantly influenced by variety and zeolite application. Zeolite-treated plants exhibited lower concentrations of all phenolic fractions compared with their respective controls, indicating an inhibitory effect of zeolites on early phenolic and flavonoid accumulation. Under control conditions, TC showed significantly higher levels of total phenols and *ortho*-diphenols than TF, whereas flavonoid levels were comparable between varieties. Despite the reduction induced by zeolite application, TC generally maintained higher absolute phenolic values than TF, suggesting a superior intrinsic antioxidant protection. In both varieties, foliar application of zeolites resulted in lower ABTS•^+^ values compared with the respective controls (*F*_3,20_ = 21.925; *p* = 1.56^-06^), although this reduction was statistically significant only in TC. In both control and zeolite-treated plants, in line with the higher basal levels of phenols, TC exhibited significantly higher ABTS•^+^ than TF, corroborating its status as a resilient variety.

At 70 DAA, variety-dependent responses to foliar zeolites became more pronounced and varied among phenolic classes. In TC, no significant differences were observed between zeolite-treated and control plants for total phenols and flavonoids, while *ortho*-diphenols were significantly reduced by zeolite application. In contrast, in TF, zeolite-treated plants exhibited significantly lower concentrations of total phenols (*F*_3,20_ = 43.418; *p* = 0.00002), *ortho*-diphenols (*F*_3,20_ = 15.062; *p* = 6.05^-09^) and flavonoids (*F*_3,20_ = 9.482; *p* = 0.0004) compared with the control.

The reduction in phenolic compounds observed in zeolite-treated plants, particularly at 42 DAA, is consistent with studies reporting lower stress-related secondary metabolites under particle films (Brito et al., 2018) or by soil zeolites application in grapevines (Cataldo et al., 2025). As phenolics are often synthesized as a protective response to UV radiation and heat (Chowdhary et al., 2022), the film, which filters part of this radiation, appears to alleviate the need for these metabolites. On the other hand, an accumulation of anthocyanins following foliar zeolite application it is described in grapes and wine due to the cooling effects and better physiological performance (Valentini et al., 2021).

Across developmental stages, total phenols and *ortho*-diphenols increased significantly in TF control plants at 70 DAA relative to 42 DAA. A seasonal increase in total phenols was also detected in TC under zeolite application, while flavonoid content increased significantly from 42 to 70 DAA across all treatments and varieties, reflecting the expected enhancement of phenolic accumulation with berry maturation and seasonal progression.

Similarly to 42 DAA, TC exhibited significantly higher antioxidant capacity (ABTS•^+^) than TF under both control and zeolite-treated conditions (*F*_3,20_ = 62.844; *p* = 2.33^-10^). In TF, zeolite-treated plants showed significantly lower ABTS•^+^ values than the control, whereas in TC no significant differences were observed between control and zeolite at this stage. Across developmental stages, significant increases in ABTS•^+^ antioxidant capacity were observed from 42 to 70 DAA in TF control plants and TC with zeolite application, suggesting an enhanced capacity to invest in antioxidant protection as summer stress accumulated. Despite the mitigating effect of zeolites, TC consistently maintained higher absolute phenolic content and antioxidant capacity than TF, reinforcing its characterization as a variety with a strong constitutive antioxidant system.

#### 3.3.2 Compatible solutes and cellular stabilization

**Figure 8:**
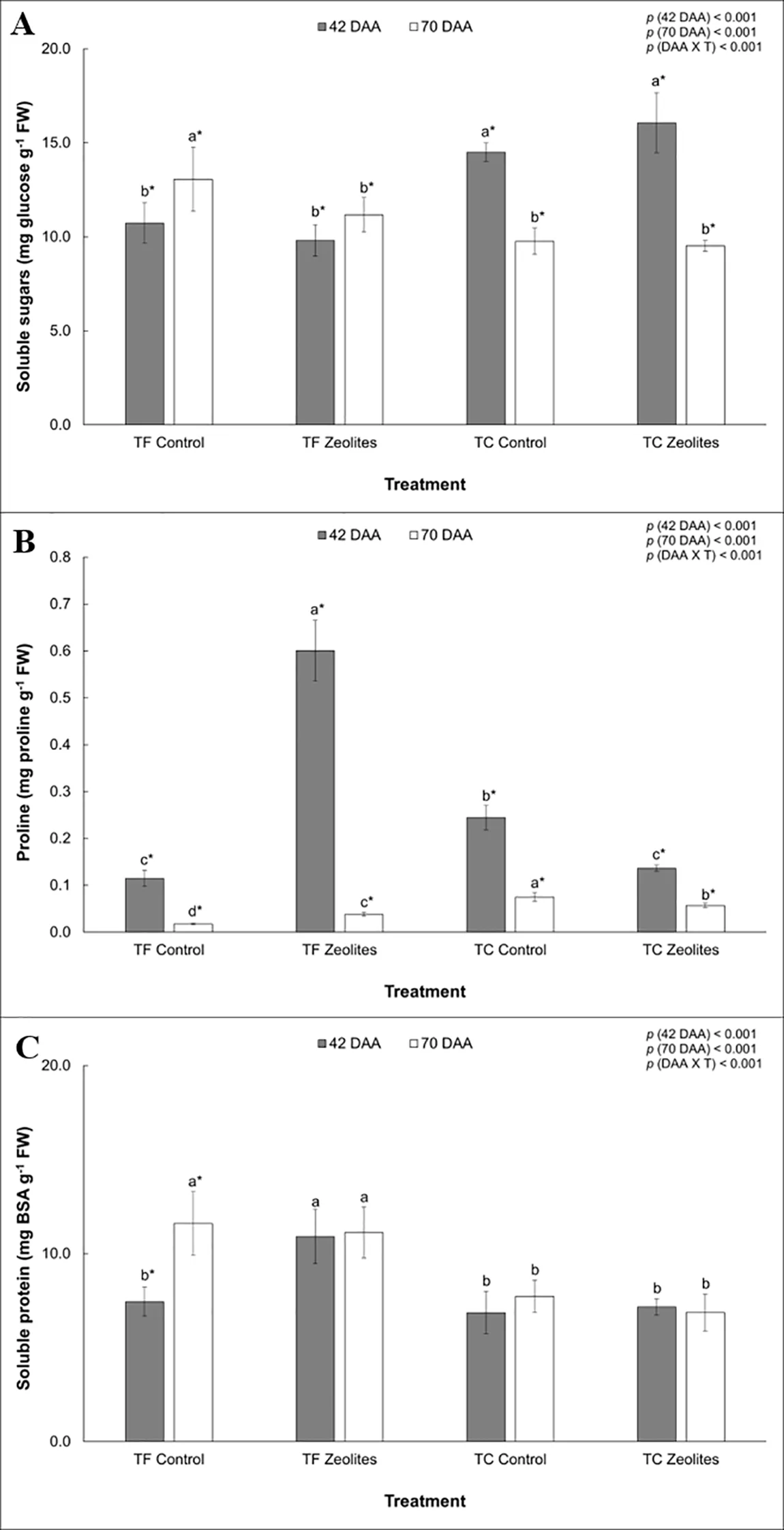
Soluble protein **(A)**, Soluble sugars **(B)** and Proline **(C)** content at 42 and 70 DAA in zeolite-treated and untreated - control plants of TF, Touriga Franca and TC, Tinto Cão varieties. Each point with vertical bars represents the average and SD, respectively. Different letters indicate significant differences between treatments, while * denote significant differences between timepoints, according Tukey’s HSD test at p < 0.05. The absence of letters and/or * indicates no significant differences among treatments and/or timepoints.

**Figure 8** presents the soluble protein, soluble sugars and proline content obtained in zeolite-treated and control plants of TF and TC varieties, at 42 and 70 DAA.

At 42 DAA soluble sugar content was significantly influenced by variety but not by zeolite application. Under control and zeolite application conditions, TC accumulated higher soluble sugar levels than TF (*F*_3,20_ = 46.332; *p* = 3.45^-09^). This suggests a superior early capacity for osmotic adjustment in TC, potentially aimed at water conservation despite its lower A at this stage. Such a mechanism is consistent with the relatively high leaf water potentials typically reported for this variety (Moutinho-Pereira et al., 2007). Zeolite application induced only minor, non-significant changes in soluble sugars, with a slight decrease in TF and a slight increase in TC.

By 70 DAA, under control conditions, soluble sugar levels were significantly higher in TF than in TC (*F*_3,20_ = 14.760; *p* = 0.00003), reversing the pattern observed at 42 DAA. At this stage, TF control plants exhibited significantly higher soluble sugar concentrations than zeolite-treated plants, whereas TC showed comparable values between treatments. This temporal reversal in soluble sugar dynamics reflects contrasting varietal vigor and carbon allocation strategies. TF displayed a progressive accumulation of soluble sugars, consistent with sustained carbon assimilation and continuous ripening, whereas TC exhibited a late-season decline, potentially associated with increased metabolic consumption under prolonged stress or earlier translocation of carbohydrates to other sinks. These contrasting sugar dynamics point to distinct metabolic priorities, with TF favoring sustained carbon accumulation, while TC appears to allocate resources preferentially toward maintenance metabolism or stress-related processes under peak summer conditions.

In turn, at 42 DAA, proline content was strongly affected by both variety and foliar zeolite application (*F*_3,20_ = 232.871; *p* = 1.01^-15^). Under control conditions, TC showed higher basal proline levels than TF, whereas under zeolite application, TF exhibited significantly higher proline concentrations than TC. At 70 DAA, in TF, proline levels remained significantly higher in zeolite-treated plants compared with the control, whereas in TC, zeolite-treated plants exhibited significantly lower proline concentrations than the control plants (*F*_3,20_ = 106.387; *p* = 1.83^-12^). These contrasting responses highlight a variety-dependent modulation of proline dynamics at later developmental stages. The differential accumulation of proline is particularly informative. In TF, zeolites induced an increase in proline accumulation, suggesting a role in osmoprotection and protein stabilization (Liang et al., 2013), further supported by the concomitant increase in soluble protein contents. In contrast, the reduction of proline levels in zeolite-treated TC plants suggests either a lower degree of osmotic stress or that osmotic adjustment is largely supported by soluble sugars, reducing the need for high proline accumulation. Similar reductions in proline induction have been reported in grapevines following soil zeolite application (Cataldo et al., 2025). In the same way, at 42 DAA, soluble protein content was significantly influenced by variety and foliar zeolite application (*F*_3,20_ = 20.712; *p* = 2.39^-06^). Under control conditions, TF and TC exhibited similar soluble protein concentrations. However, under zeolite treatment, TF showed significantly higher protein levels than TC, reflecting a marked protein accumulation in TF in response to the treatment. In TF, foliar zeolite application resulted in a marked increase in soluble protein levels compared with the respective control, whereas in TC soluble protein content remained similar between treatments at 42 DAA, indicating a limited biochemical response at this stage. At 70 DAA, TF plants exhibited significantly higher protein levels than TC under both control and zeolite-treated conditions (*F*_3,20_ = 21.284; *p* = 1.95^-06^), while no significant treatment-related differences were observed within each variety. At this latter stage, soluble protein concentrations increased significantly in TF control plants, relative to 42 DAA, reaching the highest values observed among all treatments, whereas TC displayed comparatively stable protein levels throughout the development.

#### 3.3.3 Photosynthetic pigments and photoprotection

The concentrations of photosynthetic pigments (chlorophyll *a*, *b*, total chlorophyll and carotenoids) in both varieties under control and foliar zeolite application are shown in **Table 3**.

**Table 3:** Contents of chlorophyll a, chlorophyll b, total chlorophyll, and carotenoids at 42 and 70 DAA in zeolite-treated and untreated-control plants of TF, Touriga Franca and Tinto Cão varieties.

DAA	Treatment	Chl *a*		Chl *b*		Total Chl		Carotenoids	
**42**	TF Control	0.635 ± 0.042	a*	0.484 ± 0.032	ab	1.12 ± 0.061	a*	0.223 ± 0.030	a*
	TF Zeolites	0.566 ± 0.029	a*	0.473 ± 0.060	ab	1.04 ± 0.086	ab*	0.216 ± 0.020	ab
	TC Control	0.586 ± 0.071	a*	0.546 ± 0.084	a	1.13 ± 0.139	a*	0.190 ± 0.032	ab
	TC Zeolites	0.474 ± 0.027	b*	0.437 ± 0.036	b	0.911 ± 0.059	b	0.185 ± 0.015	b
	***p value***	< 0.001		0.027		0.002		0.042	
**70**	TF Control	0.438 ± 0.043	c*	0.388 ± 0.067		0.826 ± 0.103	b*	0.166 ± 0.026	b*
	TF Zeolites	0.494 ± 0.047	bc*	0.390 ± 0.039		0.884 ± 0.068	ab*	0.221 ± 0.032	a
	TC Control	0.503 ± 0.041	b*	0.378 ± 0.029		0.881 ± 0.049	ab*	0.172 ± 0.023	b
	TC Zeolites	0.592 ± 0.022	a*	0.379 ± 0.053		0.971 ± 0.070	a	0.185 ± 0.017	ab
	***p value***	< 0.001		n.s.		0.025		0.005	
***p value (DAA X T)***		< 0.001		n.s.		< 0.001		0.018	

Values are presented as means ± SD. The results show differences between treatments and the interaction between treatment and DAA. Different letters indicate significant differences between treatments, while * denote significant differences between timepoints, according Tukey’s HSD test at p < 0.05. The absence of letters and/or * indicates no significant differences among treatments and/or timepoints.

At 42 DAA, differences between varieties were observed only for chlorophyll *a*, in zeolite-treated plants, where TF presented significantly higher concentration. At this stage, chlorophyll *a* (*F*_3,20_ = 12.950; *p* = 0.00006) and total chlorophyll (*F*_3,20_ = 3.874; *p* = 0.002) concentrations were similarly high in both control treatments, whereas foliar zeolite application induced a pronounced reduction in TC, with TF remaining largely unaffected, suggesting a clear variety-dependent response. A comparable pattern was observed for chlorophyll *b* (*F*_3,20_ = 3.874*; p* = 0.002), which was significantly reduced by zeolite application in TC but showed no significant changes in TF. Regarding carotenoids (*F*_3,20_ = 3.292*; p* = 0.042), control treatments tended to display higher concentrations than zeolite-treated plants; however, despite this tendency, particularly evident in TC, differences between treatments were not significant. The relative maintenance of carotenoid levels supports the activation of photoprotective mechanisms, such as the xanthophyll cycle to dissipate excess light energy (Latowski et al., 2011). Overall, at 42 DAA, foliar zeolite application was associated with a reduction in photosynthetic pigments, with this effect being more pronounced in TC.

At 70 DAA, variety-dependent differences were observed in chlorophyll *a* concentrations (*F*_3,20_ = 15.729*; p* = 0.00001), under both control and zeolite application conditions, with TC consistently exhibiting the highest values. At this timepoint, trends differed from those observed earlier in the season. Zeolite-treated plants of both varieties, particularly TC, showed higher chlorophyll *a* and total chlorophyll levels than control plants. This response may be associated with stress progression, as prolonged water stress can induce depletion of photosynthetic reaction centers (i.e., chlorophyll *a*) (Cataldo et al., 2025). Chlorophyll *b* showed no significant differences among treatments. For carotenoids, zeolite application significantly increased concentrations in TF (*F*_3,20_ = 5.779*; p* = 0.005), while no treatment-related differences were detected in TC.

Across developmental stages, chlorophyll *a* and total chlorophyll generally decreased from 42 to 70 DAA in control plants, reflecting natural and seasonal decline of photosynthetic pigments. Under zeolite application, this decline was attenuated or partially reversed, particularly in TC. For carotenoids, a significant temporal reduction was observed only in TF control plants. The attenuation of chlorophyll content reduction and the increase in carotenoids under zeolite application, especially at 70 DAA, suggest a delay in heat-induced leaf senescence and the activation of photoprotective mechanisms, consistent with a “stay-green” effect that may contribute to the maintenance of photosynthetic capacity during late-summer stress.

### 3.4 Leaf anatomical responses to zeolite application

Zeolites treatment did not induce significant changes in leaf protection tissues, namely the thickness of the adaxial and abaxial cuticle or the lower epidermis (**Table 4**). On the other hand, the mesophyll tissues were highly responsive to zeolite application. The treatment was associated with a significant reduction in palisade (*F*_1,23_ = 14.172*; p* = 0.001) and spongy parenchyma thickness (*F*_1,23_ = 7.794*; p* = 0.011) in both varieties, compared with the control. These reductions corresponded to approximately 11.6% and 7.2% in TF and 18.5% and 14.2% in TC, respectively. This anatomical response is likely associated with changes in leaf light and thermal microclimate induced by the mineral particle film, whose reflective properties reduce solar radiation absorption, lower leaf temperature and create a mild shading effect (Glenn, 2012; Brito et al., 2018). Under these conditions, the development of thinner mesophyll may represent an adaptative adjustment, allowing the leaf to optimize investment in photosynthetic tissues while maintaining efficiency.

**Table 4:** Leaf anatomical traits in leaves of zeolite-treated and control plants of TF, Touriga Franca and TC, Tinto Cão varieties, at 70 DAA, days after application.

DAA	Treatment	UC	UE		UPP	SP	LE	LC	MT	LT	PP/SP ratio
70	TF Control	2.20 ± 0.213	18.9 ± 2.35	a	62.0 ± 8.89	78.1 ± 9.27	12.4 ± 3.52	1.79 ± 0.142	140.1 ± 12.2	0.805 ± 0.146	175.4 ± 9.76
	TF Zeolites	2.25 ± 0.122	15.4 ± 2.69	b	54.8 ± 3.94	72.5 ± 3.88	10.3 ± 3.09	1.81 ± 0.157	127.3 ± 6.00	0.758 ± 0.061	157.1 ± 6.69
	TC Control	2.21 ± 0.233	18.3 ± 2.30	a	57.4 ± 4.51	94.1 ± 11.0	10.4 ± 1.96	1.84 ± 0.242	151.5 ± 13.1	0.615 ± 0.068	182.7 ± 13.2
	TC Zeolites	2.26 ± 0.252	16.8 ± 1.45	b	46.8 ± 4.37	80.7 ± 7.33	11.1 ± 3.39	1.68 ± 0.138	127.5 ± 7.80	0.584 ± 0.088	160.9 ± 12.5
	*p value*	n.s.	0.014		n.s.	n.s.	n.s.	n.s.	n.s.	n.s.	n.s.

Values are presented as means ± SD (n=6). Different letters indicate significant differences between treatments, according Tukey’s HSD test at p < 0.05. The absence of letters indicates no significant differences among treatments. Leaf tissues thickness is expressed in micrometers (µm). UC, upper cuticle; UE, upper epidermis; UPP, upper palisade parenchyma; SP, spongy parenchyma; LE, lower epidermis; LC, lower cuticle; MT, Mesophyll total; LT, total leaf thickness; PP/SP, palisade-to-spongy parenchyma ratio.

Consequently, total mesophyll thickness was significantly lower (*F*_1,23_ = 19.433*; p* = 0.0002) in the treated leaves, which lead to a significant decrease in the total leaf thickness, with reductions between 9.1% and 15.8%. Reductions in mesophyll thickness, particularly in palisade parenchyma have been associated with acclimation to lower irradiance, translate into a more efficient strategy for resource management (Rotondi et al., 2021; Petoumenou, 2023; Théroux-Rancourt et al., 2023). Although the literature addressing the effects of zeolites on internal leaf anatomy remains limited, the response observed in this study is consistent with that reported for other reflective particle films, such as kaolin, which reduce leaf temperature and excessive radiation without compromising photosynthetic performance in grapevine and other Mediterranean crops (Rosati et al., 2007; Dinis et al., 2018; Petoumenou, 2023; Cataldo et al., 2025).

Intrinsic varietal differences were also evident, independently of the treatment. TF and TC differed significantly in mesophyll thickness and in the palisade/spongy parenchyma ratio (PP/SP ratio), reflecting inherent differences in leaf architecture between varieties. Although both varieties generally responded similarly to zeolite application, a significant variety × treatment interaction was detected for upper epidermis thickness (*F*_1,23_ = 5.602*; p* = 0.014), reflecting a more pronounced reduction in TF (18.5%) compared with TC (8.2%).

Collectively, the results of this study suggest that foliar-applied zeolites may contribute to mitigate summer heat stress in Mediterranean grapevines, while indicating distinct variety-specific responses. By simultaneously modulating leaf temperature, photochemical efficiency, gas exchange, biochemical composition, and leaf anatomy, zeolites effectively reduce thermal load and optimize physiological performance under drought conditions. The interplay between these physiological and biochemical processes suggests an integrated adaptive response, where structural adjustments, photoprotective energy dissipation, and osmotic regulation collectively reinforce stress resilience. Despite intrinsic differences between TF and TC, the response to zeolite application was consistent across varieties, highlighting the robustness of the observed effects and suggesting that foliar zeolite application may induce adaptive mechanisms, irrespective of varietal-specific leaf architecture. These results underscore that the efficacy of particle films depends not only on the environmental stress context but also on inherent varietal traits.

## 4 Conclusions

Zeolite foliar application seems to mitigate heat stress in both Touriga Franca and Tinto Cão grapevines, as evidenced by reduced leaf temperature and enhanced photochemical efficiency, through distinct variety-specific mechanisms. Touriga Franca exhibited greater physiological and biochemical plasticity, with improved electron transport, gas exchange, and accumulation of soluble sugars, proline, and proteins under zeolite treatment, reflecting an active and dynamic acclimation to seasonal stress. In contrast, Tinto Cão displayed inherently higher phenolic content, antioxidant capacity, and a more conservative water-use strategy, with zeolite application primarily supporting photoprotection and delaying the seasonal decline of photosynthetic pigments. These contrasting responses highlight the importance of integrating short-term agronomic mitigation strategies, such as foliar zeolite application, with long-term varietal selection to enhance vineyard resilience under climate change scenarios.

Thus, the results of the present study suggest that the combined use of particle films and variety-specific adaptive traits represents a promising and innovative approach to sustain grapevine physiological performance, maintain metabolic balance, and support productivity under Mediterranean climate stress.

## Data Availability

The raw data supporting the conclusions of this article will be made available by the authors, without undue reservation.
