# Correction: Differential physiological and biochemical modulation of two grapevine varieties by foliar zeolite application under Mediterranean summer stress conditions

**DOI:** 10.3389/fpls.2026.1993494

**Published:** 2026-09-18

**Authors:** Sandra Martins, Miguel Baltazar, Cátia Brito, Ana Monteiro, Zélia Branco, Ricardo Santos, Renato Dinis, Fábio Tavares, Sandra Pereira, Lia-Tânia Dinis

**Affiliations:** 1 CITAB - Centre for the Research and Technology of Agro-Environmental and Biological Sciences, University of Trás-os-Montes and Alto Douro (UTAD), Vila Real, Portugal; 2 Inov4Agro - Institute for Innovation, Capacity Building and Sustainability of Agri-Food Production, University of Trás-os-Montes and Alto Douro (UTAD), Vila Real, Portugal

**Keywords:** climate change, long-term resilience, particle film, sustainable strategies, Tinto Cão, Touriga Franca

In the published article, the content of the **Funding** statement and the content of the **Acknowledgments** statement were incorrect. The correct **Funding** statement reads:

“The author(s) declared that financial support was received for this work and/or its publication. This work is supported by National Funds by FCT – Portuguese Foundation for Science and Technology, under the projects UID/04033/2025: Centre for the Research and Technology of Agro-Environmental and Biological Sciences (https://doi.org/10.54499/UID/04033/2025) and LA/P/0126/2020 (https://doi.org/10.54499/LA/P/0126/2020).”

The correct **Acknowledgements** statement reads:

“The authors acknowledge the project “ViTex: Valorização Integrada de Resíduos Vitivinícolas para a Sustentabilidade Agrícola”, funded by the European Regional Development Fund (ERDF), through the Incentive System for Research and Development, under the Operational Programme for Competitiveness and Internationalisation of Portugal 2030. The authors also thank the project STrengthS4WineChaiN-Scientific and Technological Synergies for Sustainable Development of the Wine Chain in the Northern Region, operation no. NORTE2030-FEDER-01786100, funded by the European Regional Development Fund (ERDF) through the Northern Regional Programme 2021-2027 (NORTE2030). The authors also acknowledge ZEOCEL Portugal Lda for providing the zeolite materials used in this study.”

The original version of this article has been updated.

